# Reversible splenial lesion syndrome associated with Graves’ disease and hepatic dysfunction: a case report

**DOI:** 10.3389/fnins.2025.1691469

**Published:** 2025-11-20

**Authors:** Xingchen He, Yijia Lin, Jing Chen, Xinyi Wu, Jindan Lv, Heng Liu, Xue Wang, Min Li, Tianyu Zhong, Yanhong Zhang, Xuliang Weng

**Affiliations:** 1 Department of Neurology, The Affiliated Guangzhou Hospital of TCM of Guangzhou University of Chinese Medicine, Guangzhou, China; 2 Sleep Research Institute of Traditional Chinese Medicine, Guangzhou Medical University, Guangzhou, China

**Keywords:** reversible splenial lesion syndrome, Graves’ disease, liver dysfunction, cytotoxic edema, case report

## Abstract

**Background:**

Reversible splenial lesion syndrome (RESLES) has been confirmed to induce severe psychiatric symptoms. This syndrome is a rare clinical condition with an etiology that remains unclear. According to current literature, the primary cause of RESLES may be associated with cytotoxic cerebral edema. First reported in 1999, reversible splenial syndrome may be triggered by bacterial or viral infections, epileptic seizures, metabolic disorders, hyperosmolar cerebral edema, and other factors. In this study, we report the case of RESLES in a patient with Graves’ disease and liver dysfunction.

**Case presentation:**

The patient was a 17-year-old female adolescent with persistent headaches, dizziness, and nausea with vomiting. Magnetic resonance imaging (MRI) suggested RESLES as the diagnosis. On admission, the patient presented with elevated free triiodothyronine (FT3) and free thyroxine (FT4), low thyroid-stimulating hormone (TSH), and positive thyroid-receptor antibodies (TRAb), meeting the diagnostic criteria for Graves’ disease. Concurrently, the levels of alanine aminotransferase (ALT) and aspartate aminotransferase (AST) were also elevated. After hormone therapy, the patient’s symptoms resolved, and imaging results returned to normal.

**Conclusion:**

This study presents a case of a patient with RESLES characterized by Graves’ disease and liver function abnormalities, who was sensitive to anti-thyroid drug (methimazole) and hormone therapy (methylprednisolone sodium succinate) and had a favorable prognosis. This study contributed to expanding the clinical understanding of RESLES and suggests that, in clinical practice, autoimmune hyperthyroidism may be a novel trigger for RESLES, while concurrent liver dysfunction in this context requires further investigation.

## Introduction

Reversible splenial lesion syndrome (RESLES), sometimes referred to as mild encephalopathy with reversible splenial lesion (MERS), is a clinical-radiological syndrome characterized by the involvement of the splenium of the corpus callosum. This syndrome was initially reported by Tada et al. (2004) and further elaborated by Garcia-Monco et al. (2011). It has various potential etiologies, including infections, epileptic seizures, withdrawal from antiepileptic drugs, autoimmune diseases, metabolic disorders, and alcohol intake. The radiological features of RESLES manifest as high signal intensity on T2-weighted imaging (T2WI), fluid-attenuated inversion recovery (FLAIR), and diffusion-weighted imaging (DWI) sequences. In contrast, isointensity is observed on T1-weighted imaging (T1WI) sequences, with no contrast enhancement (Cho et al., 2007). With timely and appropriate treatment, these radiological abnormalities can be completely resolved. The most common symptoms of RESLES include headaches, psychiatric disturbances, altered states of consciousness, and epileptic seizures (Hu et al., 2020; Feraco et al., 2018). The disease is rare and lacks specific clinical manifestations. Its etiology is complex, and cases caused by thyroid dysfunction are rarely reported in the literature, with only Xu and De Greef et al. documenting similar instances (De Greef et al., 2014; Xu et al., 2019). Moreover, there have been no international reports of reversible splenial lesion syndrome characterized by Graves’ disease with liver function abnormalities; this study reports one such case.

## Case description

**Figure 1:**
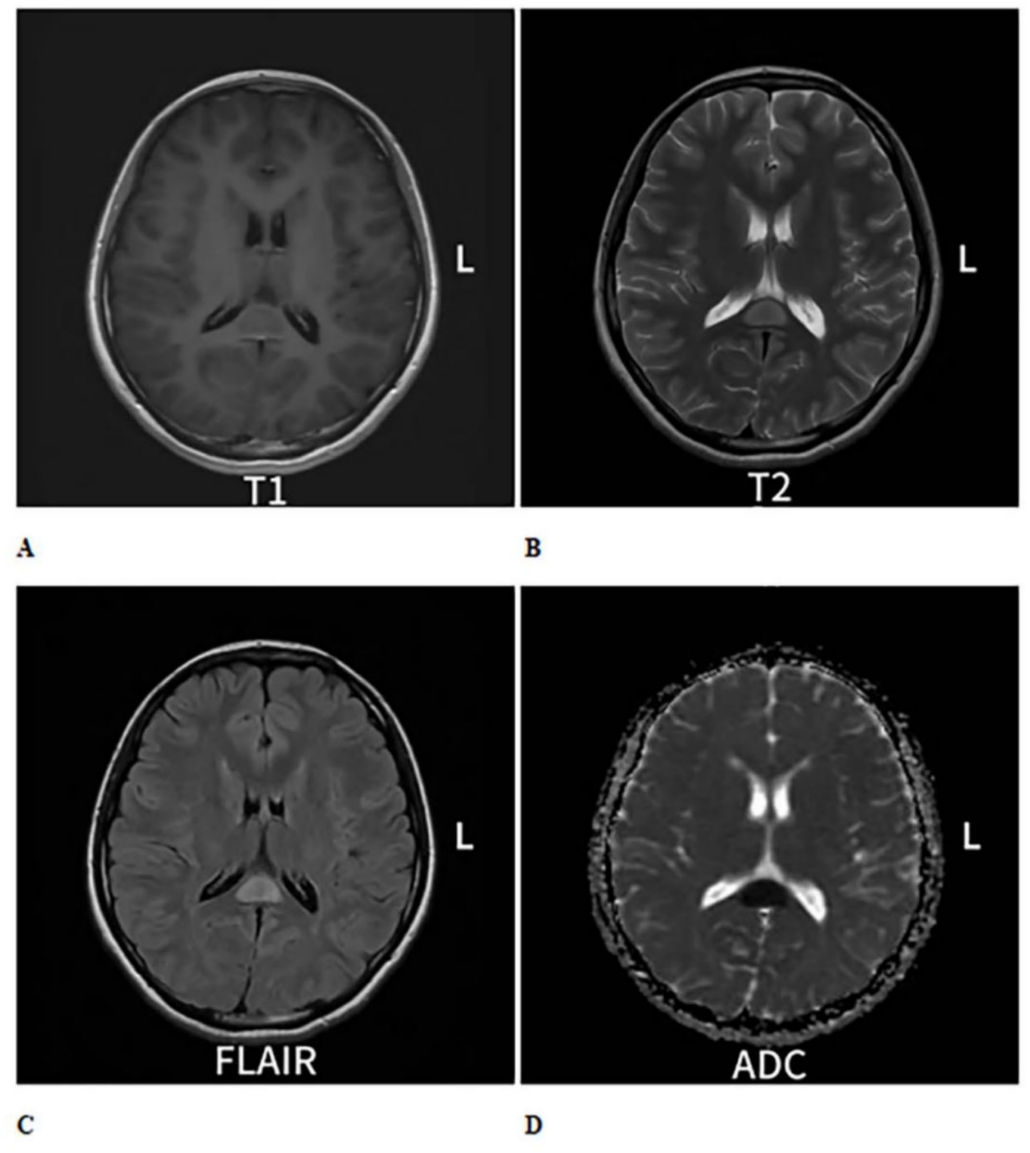
Cranial MRI plain scan with enhancement shows an oval-shaped abnormal signal in the midline area of the splenium of the corpus callosum. **(A)** On T1-weighted imaging (T1WI), it exhibits isointense to slightly hypointense signals **(B)**, while on T2-weighted imaging (T2WI), it shows hyperintense signals. **(C)** On T2 fluid-attenuated inversion recovery (T2-FLAIR), the signal is also hyperintense. **(D)** Diffusion-weighted imaging (DWI) indicates restricted diffusion within the lesion, and no significant change is observed on enhanced scans.

**Figure 2:**
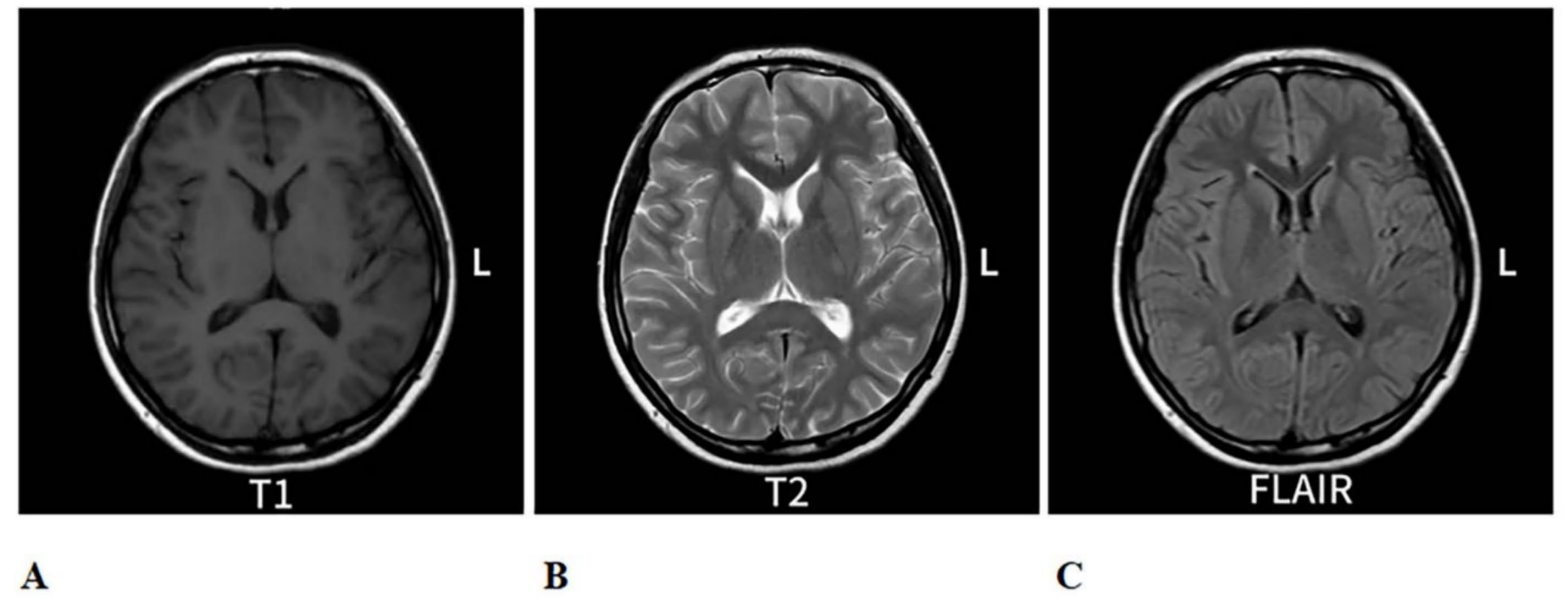
Re-evaluation of cranial MRI on 6 August 2024, compared to the MRI from 25 July 2024: The abnormal signal in the splenium of the corpus callosum. **(A)** is a T1-eighted image with low contrast. **(B)** is a T2-weighted image showing higher ontrast, highlighting fluid. **(C)** is a FLAIR image with intermediate contrast, uppressing fluid signals. Each scan shows axial brain views with symmetrical features.

A female patient with a chief complaint of “headache with dizziness for over a month” visited the Affiliated Traditional Chinese Medicine Hospital of Guangzhou Medical University on 18 July 2024. The patient had continuous headache without obvious cause since June 2024 in the frontal and temporal regions of the brain. The headache was accompanied by non-rotational dizziness. She had visited a local hospital where a head CT was performed, and it showed no significant abnormalities; the specific report was not provided. Symptomatic treatment failed to significantly improve her symptoms, leading to her referral to our hospital on 18 July. Upon admission, the patient was conscious and in a fair mental state, with a continuous headache primarily in the frontal and temporal regions, exacerbated by activity and alleviated by bed rest. Severe headaches were accompanied by nausea and non-projectile vomiting of gastric contents, along with dizziness and no symptoms of visual rotation, blurred vision, hearing loss, mobility impairment, difficulty swallowing, fever or chills, coughing or expectoration, and obvious ocular symptoms; limb weakness; and/or occasional coughing while drinking. Palpitations were noticeable; appetite was normal; sleep was poor; stools were loose, indicating a tendency toward diarrhea; and urination was normal. Upon physical examination, she appeared conscious, coherent, and cooperative. The patient’s memory, comprehension, and executive functions were normal. Both pupils were equal in size and round, about 3 mm in diameter, with intact light reflexes and absence of nystagmus, diplopia, proptosis, or periorbital edema. Ocular movements were normal, bilateral forehead wrinkles and nasolabial folds were symmetrical, tongue protrusion was centered, no atrophy or tremor of the tongue muscles was observed, the gag reflex was diminished, and the uvula deviated to the left. The thyroid was enlarged to grade II, with audible vascular bruits. Proximal limb muscle strength was grade IV, and distal limb muscle strength was grade V. The muscle tone was normal. Bilateral alternating motion, finger-nose, Romberg’s, and bilateral lower limb heel–knee-tibia tests and sensory examination revealed no abnormalities. Deep tendon reflexes were normoactive and symmetric in all four limbs. The plantar reflexes were flexor bilaterally. Stellwag’s, Joffroy’s, and von Graefe’s signs were positive, while Mobius’s sign was negative. The neck was soft, with no resistance or signs of meningeal irritation. After admission, relevant head MRI (Figure 1), thyroid function panel, and liver and kidney function tests were performed, along with other tests. The thyroid function test results were as follows: free triiodothyronine >32.55 pg./mL (reference range 2.00–4.40); free thyroxine >7.77 ng/dL (reference range 0.93–1.70); ultra-sensitive thyroid-stimulating hormone <0.005μIU/ml (reference range 0.270–4.200); thyroglobulin antibody, 282.20 IU/mL (reference range ≤115.00); and thyroid stimulating hormone receptor antibody (TRAB) > 40.00 IU/L (reference range ≤1.90). Liver function test results were as follows: alanine aminotransferase, 76.0 U/L (reference range 7.0–40.0); aspartate aminotransferase, 44.0 U/L (reference range 13.0–35.0); alkaline phosphatase, 112.00 U/L (elevated); glutathione reductase, 89.5 U/L (elevated); and total protein, 64.2 g/L (decreased). Other tests included complete blood count, urinalysis, stool routine, electrolytes, coagulation function, preoperative triad, hepatitis B and rheumatic immune panels, alpha-fetoprotein measurement, antinuclear antibody measurement, liver fluke IgG antibody test, antimitochondrial antibody examination, cerebrospinal fluid biochemistry, cerebrospinal fluid cryptococcus neoformans and tuberculosis smear examination, cerebrospinal fluid culture and identification panel, vasculitis examination, autoimmune liver disease antibody examination, serum ceruloplasmin measurement, chest digital radiography (DR), abdominal ultrasound, and lumbar spine MRI—all of which showed no significant abnormalities. Upon admission (18 July), the patient was not immediately administered antithyroid drugs due to abnormal liver function, and hepatoprotective agents were initiated for liver support. Following the evaluation of cranial magnetic resonance imaging (MRI) results and a comprehensive review of the patient’s medical history, the clinical team considered RESLES of the corpus callosum secondary to Graves’ disease as a probable diagnosis on 19 July. Consequently, antithyroid therapy with methimazole (10 mg once daily) was incorporated into the treatment regimen. Subsequently, the patient developed a severe headache accompanied by vomiting on 24 July, prompting the initiation of corticosteroid therapy. The protocol consisted of intravenous methylprednisolone sodium succinate (500 mg daily for 3 consecutive days), followed by a tapered regimen (125 mg daily for the subsequent 3 days), ultimately transitioning to oral methylprednisolone tablets with gradual dose reduction. This therapeutic strategy aimed to address neuroinflammatory complications associated with RESLES while monitoring hepatic parameters and thyroid function during the treatment course. After a period of 12 days of hepatoprotective therapy and observation, and following the initiation of corticosteroids, a multidisciplinary decision was made to commence a low dose of methimazole (10 mg once daily) on 30 July to control the underlying thyrotoxicosis. The patient’s condition gradually improved in early August, with a significant reduction in headache and resolution of dizziness, nausea, or vomiting. On 6 August 2024, a follow-up head MRI (Figure 2) was performed to compare with the MRI on 19 July 2024, which revealed no abnormality in the splenial part of the corpus callosum and illustrated a slight reduction in abnormal signals next to the posterior part of the right lateral ventricle. The key events of the patient’s clinical course, diagnostic findings, and management are summarized in Table 1.

**Table 1:** Clinical timeline, diagnostic findings, and management of the patient.

Date	Clinical event	Symptoms and signs	Diagnostic findings	Management
June 2024	Symptom Onset	Headache, dizziness	Head CT scan showed no significant abnormalities	Symptomatic treatment at local hospital (ineffective)
18 July 2024	Admission	Persistent headache, dizziness, nausea, vomiting, palpitations	Thyroid function: Elevated FT3, FT4, TRAb; Low TSH. Liver function: Elevated ALT, AST, ALP, GR; Low TP. Head MRI: Confirmed RESLES.	Hepatoprotective therapy initiated.
19–23 July 2024	Diagnosis Confirmed	Persistent headache, dizziness, nausea, vomiting, palpitations, loose stools	Diagnosis: RESLES secondary to Graves’ disease.	Hepatoprotective therapy continued.
24 July 2024	Clinical Worsening	Severe headache with vomiting	No significant abnormalities in other tests.	Hepatoprotective therapy continued.
25 July 2024	Corticosteroid Initiation	Severe headache with vomiting	Cerebrospinal fluid analysis unremarkable.	IV methylprednisolone (500 mg/day × 3d, then tapered).
30 July 2024	Antithyroid Therapy Start	Headache alleviated	Hepatic enzymes improved.	Methimazole 10 mg/day started.
Early August 2024	Clinical Improvement	Headache, dizziness, nausea, vomiting resolved	Thyroid function improved.	Methimazole continued; oral methylprednisolone tapered.
6 August 2024	Follow-up	Asymptomatic	Head MRI: Complete resolution of splenial lesion.	Discharge planned.

## Discussion

RESLES is a clinical–radiological syndrome primarily affecting the splenium of the corpus callosum, characterized by the presence of ovoid, non-enhancing lesions in the splenial region of the corpus callosum on magnetic resonance imaging (MRI), which can completely resolve over time (Renard et al., 2014). The clinical manifestations of RESLES typically include neurological symptoms such as headache, dizziness, nausea and vomiting, and altered consciousness. In reports by Xu et al. (2021), there have also been cases with fever as a precursor symptom. Clinically, it lacks specificity and requires differentiation from a range of other conditions, including demyelinating diseases such as acute disseminated encephalomyelitis (ADEM) and multiple sclerosis (MS), as well as specific entities like corpus callosum infarction and Marchiafava-Bignami disease (MBD). Additionally, lymphomas and other disorders that present with alien hand syndrome (AHS) should also be considered in the differential diagnosis (Supplementary Table S1). (A detailed comparison of the clinical and radiological features distinguishing RESLES from its mimics). Extensive research (Starkey et al., 2017; Prilipko et al., 2005) highlights that RESLES is associated with the interplay of excitotoxicity, energy metabolism disorders, ion pump failure, and imbalances in water and electrolytes inside and outside cells, leading to cytotoxic cerebral edema. The pathogenesis of RESLES in Graves’ disease is closely linked to gliocyte metabolic disorders caused by thyroid toxicity. TRAb constantly activates TSH receptors, causing a surge in thyroid hormones (Bartalena, 2013). As a result, free T3, transported efficiently by monocarboxylate transporter 8 (MCT8), crosses the blood–brain barrier and targets astrocyte N-methyl-D-aspartate (NMDA) receptors (Wirth et al., 2014). Overactivation of this NMDA receptor triggers calcium-dependent mitochondrial dysfunction, reducing Na+/K + -ATPase activity, which disrupts osmotic balance in neuron–glia units, causing cytotoxic edema (Zheng et al., 2023). The corpus callosum splenium, metabolically vulnerable, is particularly susceptible (Blaauw and Meiners, 2020). When thyroid toxicity drastically reduces ATP synthesis, these high-metabolic-demand tissues face endoplasmic reticulum stress, hindering the synthesis of myelin basic protein. However, this damage may be reversible. After thyroid function normalizes, astrocyte aquaporin-4 (AQP4) regulation can resolve edema, and rapid oligodendrocyte precursor cell migration can fulfill myelin repair needs (Zhang et al., 2015). Currently, the therapeutic approach for this condition predominantly involves the management of the underlying primary disease and the administration of corticosteroids. Additionally, evidence from some research studies suggests that plasmapheresis may serve as an alternative treatment option for patients (Varol et al., 2022). RESLES has been reported worldwide, mostly caused by infections, epilepsy, and antineoplastic drugs. However, RESLES due to thyroid disease is deemed a rare type with just a few case reports. De Greef et al. reported a case with only elevated anti-thyroid antibodies (anti-TPO), but without the overt hormonal imbalance or clinical thyrotoxicosis characteristic of Graves’ disease. The mechanism in that context was postulated to involve a pure autoimmune cascade. Xu et al. reported cases associated with subclinical thyroid dysfunction, including both subclinical hyper- and hypothyroidism, where patients typically have minimal or no symptoms. Unlike Xu et al.’s report on RESLES, this study confirmed a direct link between Graves’ disease (rather than simple hyperthyroidism) and RESLES. TRAb, as TSH receptor-stimulating antibodies, at high levels (>40 IU/L) may permeate the blood–brain barrier, activate TSH receptors in the central nervous system, and cause neuroglial dysfunction, offering new mechanistic research directions. The patient in the study is a 17-year-old female with no epilepsy or infection (viral or bacterial), presenting with persistent headache, dizziness, and hypermetabolic symptoms like palpitations and irritability. She had a grade II goiter, elevated FT3, decreased FT4 and TSH, and positive TRAb, meeting the diagnostic criteria for Graves’ disease. Concurrently, the patient’s head MRI during admission indicated a lesion in the splenial region of the corpus callosum; therefore, we believe that the patient’s condition was a reversible splenial lesion caused by hyperthyroidism. Notably, the patient had no underlying diseases or medication history, and ancillary tests excluded factors like infection (viral hepatitis), non-alcoholic fatty liver disease (NAFLD), metabolic-associated fatty liver disease (MAFLD), gallstones, and genetic defects. However, post-admission liver function tests showed elevated transaminases, likely due to liver metabolic overload from Graves’ disease. Although liver dysfunction and RESLES are temporally related, and liver dysfunction could potentially cause RESLES, especially with metabolic or autoimmune issues, the causality remains speculative. In addition to the metabolic overload from hyperthyroidism, we also considered other potential causes of liver injury, such as autoimmune hepatitis overlap or drug-induced liver injury. However, these were deemed less likely in this case. Autoimmune hepatitis was largely excluded by negative results in the autoimmune liver disease antibody panel. Drug-induced injury was unlikely, as the liver dysfunction was present prior to the initiation of methimazole and steroid therapy. Therefore, while not definitively proven, Graves’ disease-related hepatic metabolic stress remains the most plausible explanation for the observed transaminitis. No reports regarding RESLES caused by Graves’ disease with concurrent liver dysfunction have been observed in databases such as PUBMED; thus, this study expands the clinical presentation of RESLES, enriches its etiology, and deepens our understanding of the condition, which is of considerable significance.

## Data Availability

The original contributions presented in the study are included in the article/Supplementary material, further inquiries can be directed to the corresponding authors.
