# Supplementary material for: Reversible splenial lesion syndrome associated with Graves’ disease and hepatic dysfunction: a case report
Source: Front Neurosci. 2025 Nov 20;19:1691469. doi: 10.3389/fnins.2025.1691469 (PMC12675446; doi:10.3389/fnins.2025.1691469)
Supplement: Supplementary file 1 [file Supplementary_file_1.docx]

****Supplementary Table 1 Differential diagnosis of RESLES****

| ****Condition**** | ****Clinical Features**** | ****MRI Features**** | ****Prognosis**** |
| --- | --- | --- | --- |
| ****RESLES**** | Headache, delirium, seizures | Reversible splenial lesion, DWI restriction | Favorable with treatment |
| ****ADEM**** | Multifocal neurologic deficits | Multifocal white matter lesions, may enhance | Variable |
| ****Metabolic Encephalopathy**** | Altered consciousness, metabolic derangement | Symmetric deep gray matter involvement | Depends on cause |
| ****MERS**** | Encephalopathy, infection-related | Splenial lesion ± white matter changes | Usually good |
